# Privileges, and Permissions: Theorising Intersectionality and Cultures of Control in the Care of People Living With Dementia in Acute Hospital Settings

**DOI:** 10.1111/1467-9566.13869

**Published:** 2025-01-03

**Authors:** Shadreck Mwale, Andy Northcott, Katie Featherstone

**Affiliations:** ^1^ Geller Institute of Ageing and Memory University of West London Ealing UK

## Abstract

A longstanding body of public enquiries and research identifies people living with dementia experience systemic inequalities within hospital settings, concluding a focus on improving care cultures is required. Drawing on a 3‐year multi‐sited hospital ethnography, this paper examines everyday cultures of care in NHS acute hospital wards to interrogate how ethnicity, gender and social class intersects to shape the care of people living with dementia. Drawing on Collins' concept of intersectionality and the relational nature of power, the analysis reveals that while cared for by diverse teams of healthcare professionals, a patients' age, ethnicity, gender and social class, as interconnected categories, influences the tightening of ward rules for some people living with dementia and the granting of significant privileges for others. Focussing on walking within the ward, with a large number of people living with dementia classified as ‘wandering’, we explore ways in which intersectional identities informed who was granted privileges to leave the bedside and ‘wander’ the ward, and who experienced further control. The paper concludes that institutional racism and attitudes to gender, social class and ageing permeate the routine organisation and delivery of care within NHS acute hospital wards to significantly impact people living with dementia, and in turn, increases the consideration of care pathways that emphasise their discharge to institutional settings.

## 1 Introduction

Globally, 55 million people are estimated to be living with dementia (WHO 2023). In the UK that number is about 944,000 and is predicted to reach 1.6 million by 2050 (ARUK 2023). However, those living with the condition experience systemic inequalities and poor outcomes and experiences across care settings.

Here we focus our analytic gaze on the acute hospital, a key institutional setting, which is a significant site of care for people living with dementia. In the UK, an estimated 70% of NHS acute admissions are older people, of which 40% are people living with dementia (Prince et al. 2014), representing a significant proportion of emergency admissions (77%), typically with treatable conditions such as pneumonia, fractures, frailty, sepsis and urinary tract infections. Thus, at any given time, approximately 25% of acute hospital admissions are also people living with dementia (Sampson et al. 2014). However, these figures may underestimate this population (Crowther, Bennett, and Holmes 2017; DoH 2014 cited in Alzheimer's Society 2016).

A body of public enquiries, reports (DoH 2006; House of Lords 2007; CQC 2011; Francis 2013; Andrews, Fellow, and Butler 2014) and research describe the acute hospital setting as ‘challenging’ (Sampson et al. 2014:194; Scerri, Innes, Scerri, Innes, and Scerri 2020) and ‘dangerous’ (Mathews, Epperson, and Arnold 2013, 465) for people living with dementia. Adverse events they experience in the acute hospital are typically associated with falls, delirium (Pan et al. 2018), incontinence (Hofmann and Hahn 2014), functional decline (Moyle et al. 2011; Thornlow, Anderson, and Oddone 2009; George, Long, and Vincent 2013) prolonged admission (Tan et al. 2014), and distress (De Bellis et al. 2013; White et al. 2017). Hospitalisation is associated with increased risk of deterioration (Goldberg et al. 2012), institutionalisation (Lehmann et al. 2018), and death (Sampson et al. 2014), in comparison to similar patients without a diagnosis of dementia. These systemic inequalities point to entrenched cultures of care within acute hospital settings (House of Lords 2007).

We are not alone in examining the experiences of people living with dementia and the care they receive in hospital. Kelly (2014) (in England) and Thorsen and Nielsen 2023 ethnography (in Denmark), examined the social and relational nature of care, and how they contour the experiences of people living with dementia in hospital. Others have questioned the meanings of personalised care in dementia care; Bailey et al. (2015) examined the emotional labour of healthcare assistants caring for patients considered a ‘challenge’. In this journal Driessen and Ibanez (2020), explored the delivery of person‐centred care in relation to food to people living with dementia within long term care settings. Similarly, Haeusermann (2018), explored how care workers negotiate the delivery of intimate care in institutional contexts. Still Scales et al. (2017) ethnography, examined power and person‐centred care in the NHS. Collectively, this body of work points to the importance of examining care cultures people living with dementia experience within this setting.

However, the significant impact of the hospital setting on people with intersectional identities especially ethnic minority patients has yet to be examined. African and African Caribbean people living with dementia are a key population at high risk of inequitable access to and poor experiences of health, social care and community services, experience failings in care and have poor health and wellbeing outcomes. This pattern of inequalities has been identified for at least 20 years. We also know that social class and gender have an impact on health outcomes (Nazroon 2003; Tolhurst, Weicht, and Runacres. 2023); however, we know little about how these intersect to inform hospital care.

### 1.1 Walking Within the Ward

Within this paper we focus on an aspect of everyday life within these wards—walking—which for people living with dementia who attempt to leave the bedside or explore the ward is typically framed by ward staff as “wandering” and a behavioural feature of dementia (Featherstone and Northcott 2020). This is the language of these wards and the wider cultures of these hospitals and is a key focus in the organisation and delivery of routine timetabled care within the contemporary acute ward.

The understandings found within these wards also reflect the conceptualisations of dementia and the perceived behaviour of people living with dementia found within the wider biomedical literature. Recognised clinically as part of a constellation of ‘neuropsychiatric symptoms’ commonly described as ‘behavioural and psychological symptoms’ (BPSD) of dementia. However, we support interpretations which recognise these ‘behaviours’ as reflecting an individual's desire to achieve autonomy (walking within the ward) (Featherstone and Northcott 2020), reflecting ongoing critiques of the use of ‘wandering’ and other terms describing aspects of behaviour (Wolverson et al. 2019, 2021).

### 1.2 Privileges in Care Settings

It is also important to consider ways in which racism and discrimination shapes approaches to care. As outlined above, care does not take place in a vacuum; rather, wider societal views permeate and contour approaches to care. Healthcare occurs within often unacknowledged historical and established institutional frameworks of racial and class hierarchies of privilege. By privilege we refer to Peggy McIntosh's (1990) concept of privilege as an ‘*invisible weightless knapsack of special provisions, maps, passports, codebooks, visas, clothes, tools, and blank checks’*. She observes that ethnicity, gender and class can afford certain people privileges in everyday life which helps them access services and go about daily life aided by these privileges. These privileges are unacknowledged and often invisible to those who benefit from them. We have written elsewhere about the reality of institutional racism experienced by people living with dementia of colour having negative impacts on care experiences and health outcomes for this population; however, there has been little examination of the underlying mechanisms or potential ways to intervene (Mwale 2023). McIntosh's concept is useful for examining practices in the care of people living with dementia and the ways in which attitudes to ethnicity, social class and gender can permeate care approaches and practices.

In addition to ethnicity, both social class and gender are considered significant in shaping healthcare experiences and outcomes. Ethnicity is known to intersect with socioeconomic status (Nazroon 2003; Karlsen & Nazroo 2002; Stopforth et al. 2023) to influence health outcomes. However, because social class is not a protected characteristic within the UK Equality Act (2010) (Walkerdine 2021), classist attitudes and discrimination in healthcare typically remain unexamined. Many attempts to address social class related discrimination have been through a focus on social mobility, with the view that moving up the social strata improves people's life chances, but to little or no effect to date. However, as hooks (2000) argues, social class matters, and its intersections with ethnicity and gender is consequential for marginalised groups (Karlsen & Nazroo 2002; Stopforth et al. 2023). However, social class, its intersections and impacts as experienced by older people living with dementia has been neglected as an issue in sociological analysis (Evers 2022; Calasanti & Slevin 2013; Karlsen & Nazroo 2002). In this journal, Tolhurst, Weicht, and Runacres (2023) lament the lack of research focus on the gendered nature of experiences of dementia, calling for a nuanced examination of the experiences of women living with dementia.

Drawing on ethnographic data examining the care of people living with dementia in NHS acute hospital settings in England and Wales, this paper examines how ethnicity, social class and gender intersects with tacit organisational rules to contour ward staff approaches to the care of people living with dementia. Specifically, it focuses on how the application of these rules is predicated on and shaped by institutionalised attitudes to ethnicity, gender, and social class. Our aim is to examine the social processes underpinning these cultures of care and their impacts on patient populations most likely to experience poor health outcomes. The following section outlines the theoretical approach underpinning this paper.

### 1.3 Theorising Intersectionality and Cultures of Control in Dementia Care in Acute Hospital Settings

Sociology has illustrated how gender, ethnicity and social class are connected to experiencing a range of social inequalities, including how these social categories intersect to produce varied and multiple health inequalities (Karlsen & Nazroo 2002; Stopforth et al. 2023).

We utilise Collins and Bilge's (2020) theory of intersectionality, which provides an examination of how intersecting power relations influence social relations. However, as a sociological concept, intersectionality has increasingly become a trope within the field of healthcare, reduced to merely indicating links between a wide range of factors associated with health outcomes, and divorced from its political foundations, which sought to illustrate the consequences of subjugation for the multiply subordinated. In this paper we reclaim its original goals.

As an investigative tool, intersectionality takes gender, social class, ability, ethnicity, age, citizenship and nation as interconnected and constantly shaping and reshaping each other (Collins and Bilge 2020; Nash 2011). It emanates from Black feminist thought, which sought to challenge the idea of universal understanding and experiences of inequality and marginalisation in which inequality and subjugation are viewed through a single‐axis framework. Rather it proposes focussing the analytical gaze on ways in which power relations (Foucault 2003) of multiple and layered categories intersect and interact. This means that while groups can all experience inequality, some individuals are worse off by virtue of belonging to multiple intersecting groups (Crenshaw 1991). The issue Black feminists highlighted was how Black women are marginalised and face oppression first as women and then due to their ethnicity (Nash 2011). Adding sexual orientation and disability further adds to their marginalisation and erasure (Collins & Bilge 2020). This expansive body of work (Collins & Bilge 2020; Crenshaw 1991; Collins 2000, 2012, 2019; Nash 2008, 2011, 2017; hooks 2000) has made significant contribution to understandings of discrimination and inequality.

Central to intersectionality is the call to move away from seeing subjugation and discrimination as emanating from singular, discrete and universal vectors, eliding those multiply burdened, but instead examine how life experiences are impacted by multiple, layered, forms of discrimination, some of which may be discreet (Butler 2002). We draw on Foucault's (2003, 2016) conception of bodies and power, where power exerted on bodies is to be understood as a political tool strategically employed for the unilateral imposition of power, which is at once productive, diffuse and varied in form. Within this paper, we apply this view of power relations to the experiences of people living with dementia in acute hospital settings. This is not to elide the multiply burdened nature of Black womanhood, but rather to draw on the tools this approach proffers to make sense of and illustrate ways in which forms of subjugation (Foucault 2016) exist for racialised and marginalised vulnerable adults within institutional care settings (Foucault 2003; Goffman 1967). Within institutional care settings (Foucault 2003), understanding experiences of people living with dementia using this framework provides space to analyse how ethnicity, gender and social class, intersect to shape their care, with dementia itself a stigmatised and silenced condition.

We are not the first to consider the significance of intersectionality in understanding dementia care. WeBel (2022) has examined the intersections of age, gender and race during diagnosis; Watchman (2018) the intersections of intellectual disability, age and dementia and Thomas and Milligan (2018) the intersections of disability rights and disablism. Others (Ma and Joshi 2022; Koehn et al. 2013; Roes et al. 2022) have examined the intersections of migration, diaspora life and care of older people, and Hulko (2016) examined the intersections of sexuality, ageing and dementia. While this body of work is significant, the majority rely on secondary literature reviews rather than primary research.

This paper is the first to draw on primary data to examine intersectional experiences of people living with dementia and how this informs their care within acute hospital settings. It contributes to debates about power relations in the organisation and delivery of care by focussing on marginalisation, power, privilege, and its impacts.

### 1.4 Institutional Cultures of Control

We do this by bringing the historical socio‐political focus of intersectionality to the fore in the analysis of the care of racialised people living with dementia and how institutionalised racist thinking permeates spaces of care. While care occurs in highly regulated contexts, it does not occur in a vacuum. Rather, it is embedded within historical and established institutional cultures that include everyday taken for granted practices and unwritten rules (Foucault 2016) that delineate appropriate behaviour and deserving patient‐hood. These also originate from established social practices and attitudes in the categorisation of people into hierarchies of need, resulting in the creation of racial categories passed on through generations and institutional practices (Monk 2022) in what Alfred Schults (1972) refers to as ‘common sense manner’. These racial and social class categories are enacted by the apportioning of privileges to those located on top of the hierarchy, while taking away rights and access to privileges to those considered to be in lesser categories. This paper illustrates how implicit power is evident in everyday institutional practice (Foucault 2016) to bring about the subjugation of racialised people living with dementia. We also examine how social categories of gender and social class influence privileges and permissions in their care.

The state has legitimised social categories, formalising, recognising, emphasising, and creating differences between perceived groups, which have over time become embedded in legal and institutional structures (Foucault 2016). These categories, including ethnicity, gender, and social class, are routine formal requirements in administrative forms, identity cards and health records (Monk 2022). In everyday life and within institutional healthcare settings, these perceived differences manifest through stereotyping and discrimination in different domains, benefiting some to the detriment of others (Massey 2007).

At the core of state legitimised categories what often goes unsaid is that their purpose is to bureaucratically create a sense of belonging, constructing the ‘other’, ‘us and them’, and ‘the deserving and undeserving’ (Jensen 2011). These categories become the basis for informing the distribution of resources, access to services, and rights. In creating racialised categories as the ‘other’, these perceived outsiders have often been presented as a threats and undesirables whose activities must be watched and controlled.

Garland's (2002) concept of cultures of control derives from his observations of developments in public policy and how best to respond and manage crime in the United States and the UK. He asserts that the social need to live in the here and now has meant that policy shifts that should have been challenged have often gone unchallenged. Garland is concerned with how contemporary criminal penal systems of governance have vested interests in control and the informal social controls entrenched in everyday activities and interactions of civil society. For Garland, the widely desired goal of controlling and reducing crime in contemporary society ‘also *entails new practices of controlling behaviour and doing justice, revised conceptions of social order and social control, and altered ways of maintaining social cohesion and managing group relations. The remodelling of an established institutional, the emerged of different objectives and priorities, and the appearance of new ideas about the nature of crime and of criminals also suggest shifts in the cultural underpinning of these institutions*’ (p. 6). He asserts that the materialisation of new responses to crime is associated with new mentalities and conceptions of the problem in hand.

Parallels can be drawn between the hospital and the prison, with the institutional need for order and compliance and conceptions of good personhood (Foucault 2003). We draw on this idea to show how the need for social order and ensuring patients comply with institutional rules brings into sharp relief the power dynamics in which ward staff control and manage patients and shape their personhood within the setting (Boddington, Northcott, and Featherstone 2024). The significant population of people living with dementia present a new challenge for health services and policymakers alike. The contemporary challenges of patients whose conditions mean they do not fit neatly into the ideal of a ‘good’ or compliant patient, and which requires reconfiguring the provision of healthcare to maintain and sustain operations within wards. In this case patient control and containment can become the default response; ‘what we do here’.

It is therefore important to draw on approaches that try to make sense of the institutional structures that facilitate cultures of control by understanding how ethnicity, gender and social class intersect within healthcare settings. In this context, we take an intersectional approach as a critical approach and praxis to examine how established power dynamics and multiple interlocking systems of power in institutional contexts are sustained across time and space (Foucault 2003; Lawrence and Buchanan 2017). It is a useful tool for analysing the logics, assumptions and practices, underpinning care and attitudes to marginalised people living with dementia in acute hospital ward settings.

## 2 Methods

Data in this paper is derived from a larger ethnographic study funded by National Institute for Health and Social Care Research (NIHR HSDR study ID TBA) examining the everyday organisation and delivery of care for people living with dementia within acute hospital wards. This ethnographic study had a specific focus on continence care for people living with dementia within these settings and reported elsewhere (Northcott, Boddington, and Featherstone 2022; Featherstone et al. 2022). Our ethnographic approach facilitated the wider examination of everyday routines, practices and behaviours within and across multi‐disciplinary healthcare professionals teams (Quinlan 2009), and the social and institutional forces contouring the delivery of hospital care (Greenhalgh and Swinglehurst 2011) for people living with dementia.

This ethnography was carried out within 3 hospitals across England and Wales (2017–2020), purposefully selected to represent average hospitals (none had been placed in ‘special measures’ or identified as exceptional by independent regulators). They all served urban and rural populations with diverse socioeconomic catchment areas. Within these hospitals, data collection focussed within 6 wards known to admit large numbers of people living with dementia for a range of acute conditions: general medical wards and medical assessment units.

### 2.1 Data Collection

Within each ward we (KF and AN) conducted 30 days of observation over a period of 8 weeks of detailed fieldwork. Observation periods included handovers, morning, day, evening and nights, including weekdays, and weekends. Observations ranged in duration from two to 6 h and were reactive to events during observation. We utilised non‐participant observation, concentrating on the visible work of nurses and health care assistants (HCAs) to explore their everyday work. This included the timetabled routines of observation rounds, personal care, medication rounds, and mealtimes within these wards. We also focussed on responses to personal alarms, calls for assistance, and decisions to prioritise or defer, to examine the classification, urgency, and management of care needs when it disrupted ward routines. We also focused on communication, language, and everyday interactions, observing handovers, multidisciplinary team meetings, admissions, and conversations with carers, all opportunities for sharing information about people living with dementia. This enabled us to provide detailed understandings of the organisational and interactional care processes that affect this patient group. Approximately 500,000 words of observational field notes were collected, written up, transcribed, cleaned and anonymised (Van Maanen 2011; Emerson, Fretz, and Shaw 2011) by the ethnographers (KF and AN).

We present a ‘thick description’ of data to provide context to allow the reader to assess the rigour of our findings (Geertz 1973). To optimise rigour (Herriott and Firestone 1983), our approach involved prolonged engagement within wards and comparisons across hospital sites (Vogt 2002) to achieve data saturation and the search for negative cases (Glaser and Strauss 1967; Saunders et al. 2018). Credibility checks included presenting emergent analysis to ward staff, people living with dementia, and their care partners for discussion. To check confirmability and interpretations of the findings, analysis was carried out by all authors.

Ethics Committee approval was granted by the NHS Research Ethics Service via the Wales Research Ethics Committee 3 [*(reference number: 15/WA/0191)*) with approval from the Health Research Authority and Health and Care Research Wales granted on 5^th^ September 2018. The research project was approved for the purposes of the Mental Capacity Act 2005 (Section 31).

### 2.2 Data Analysis

Within this paper we utilised ethnographic abduction (Atkinson 2014) to draw out analytic ideas from the observations to ensure our analysis reflects the complexity of everyday life. This approach supports an interpretative process that is both explorative and grounded in data (Glaser and Strauss 1967). At this stage we (KF, AN and SM) re‐examined the raw data with a focus on social differences and their connections with marginalisation.

Strategies used for the development and testing of analytic concepts and categories, included the careful reading of the data, looking for patterns and relationships, noting anything surprising and inconsistencies and contradictions across the perspectives gathered. Line‐by‐line coding is not appropriate for field notes; instead, coding involved whole events or scenarios (Suddaby 2006). Initially, this produced a collection of “sensitising concepts” (Vogt 2002) and analytic memos, which informed the development of more refined and stable analytic concepts. The analytic concepts that emerged from this process were then further tested and refined to develop robust analytic concepts that transcend local contexts to identify broader structural conditions (Atkinson 2014) influencing care.

To optimise rigour (Herriott and Firestone 1983) our approach emphasised comparisons across sites (Vogt 2002) and carrying out prolonged engagement with these wards, to achieve data saturation and the search for negative cases (Glaser and Strauss 1967; Saunders et al. 2018). To check confirmability and interpretations of the findings, the analysis was carried out by all authors. Credibility checks included presenting emergent analysis to a steering group of older people, people living with dementia and their care partners.

The presentation of findings utilises an ethnographic “thick description” of data to provide readers with ways to connect concepts, policies, and practice to detailed empirical examples. This approach allows the reader to connect to the social world of these wards and how this connects with wider issues of care and subjugation within institutional settings (Glaser and Strauss 1967; Saunders et al. 2018).

### 2.3 Reporting Practices

We refer to the diagnosis, race/ethnicity and sociodemographic characteristics of our participants throughout this article. These descriptions are taken from the records within these wards, including via handover from the nurse in charge, descriptions of patients within handover documents and the semi‐public admissions board during each shift observed. We are aware that approaches utilising external determinations of patient physical characteristics by ward teams, rather than self‐reported from participants themselves, has the potential to perpetuate racism. However, this reflects the recording practices and language of these wards; diagnostic and identity assessments could quickly be made by staff, become attached to an older person within their records and remain uncontested (Flanagin, Frey, and Christiansen. 2021). For anonymity, we have given all participants pseudonyms.

### 2.4 Findings: Rules of the Ward and Assigning Privileges: Intersections of Ethnicity, Social Class and Gender

We identified that within these wards the importance placed on containing people living with dementia within the bed or within the chair at the bedside could also be informed by embedded bias. These approaches to care were typically embedded within these wards' organisational cultures of care, often rationalised as a response to the perceived behaviours (resistance to timetabled care and behaviour viewed as disruptive, inappropriate, or transgressive) of people living with dementia (Featherstone et al. 2019) or indeed staff concerns about the risks of falls. Within all of these wards, we identified cultures of control including the routine use of a range of controlling and containing practices, verbal commands, the use of side rooms and bedsides as spaces for and means for limiting patient movements. These organisational approaches were used primarily to support the delivery of routine bedside care, including medications, mealtimes, personal care, and continence care for people living with dementia. We identified that for people living with dementia with intersectional identities, these practices were intensified. For wider context of these ward settings see Featherstone and Northcott's (2020) work.

### 2.5 Intersections of Ethnicity and Dementia

We identified intersections of ethnicity and dementia that increased the urgency and anxiety underlying the approaches ward staff used in the organisation and delivery of bedside care and how they responded to the care needs of people living with dementia. These intersected with a patient's identity of being a person living with dementia in ways that could additionally inform the privileges they experienced within these wards and intensified staff beliefs about danger, risk, and the surveillance they required, and in turn experienced.

We found that ethnicity and specifically being identified as African Caribbean, Asian or from an ethnic minority could inform ward staff responses. Tolerance appeared to decrease and fear and specifically the fear of violence, appeared to increase. In this ward, 4 men (we have given then pseudonyms David, Michael, Edward and John) who all have a dementia diagnosis, described by the ward team as ‘unpredictable’ and ‘aggressive’, and all were closely supervised. Their care was also governed by ‘Deprivation of Liberty Safeguards’ (DoLS), a formal legal framework introduced in 2009 designed as a safeguard to ensure that no one is inappropriately or arbitrarily deprived of their liberty. Across the shifts, these patients were observed as having access to different privileges of where they are permitted to go and what they are permitted to do within this ward.

John is an African Caribbean man described by the ward team as ‘unpredictable’, ‘aggressive’ and a ‘big man;’ he has a security guard (rather than the usual one‐to‐one agency healthcare assistant (HCA)) sitting with him in his single occupancy room. Whenever he leaves the room, the security guard shadows him and stays close at his side with his arms folded.

9am, I walk down the ward with one of the nurses and we meet John. He recognises her and they say hello, but the security guard immediately calls him back into his room. He remains in his single occupancy room sitting in the chair with the security guard sitting next to him, who is silent with his arms folded.

A while later he is standing in the corridor with the security guard, who is standing just behind him with his arms folded. The meaningful activities coordinator smiles and leads John by the hand into the activities room. She also says hello to the silent security guard standing behind him and shakes his hand asking John: Is he your mate? He spends the afternoon with the meaningful activities co‐ordinator.

(site 2 day 1)

Staff descriptions and designation of John as ‘unpredictable’ is in keeping with Jackson's (2020) observations about how racial categories have brought about the bestialization of Black people as dangerous and thus require controlling and watching. Contrast this with staff approaches during the same shift to David, categorised as White and also described as ‘unpredictable’ by the ward team and assigned a one‐to‐one agency healthcare assistant (HCA). David is able to walk around the ward unchallenged with the one‐to‐one agency HCA shadowing him at a distance. He is even permitted to stand at the nurses station and mix‐up the neatly stacked forms and store of dressings.

David is wearing a red pyjama top that is his own, tucked into green hospital pyjama bottoms pulled up very high. He has paper and pen in his top pocket, and walks slowly back to the bay, the 1 to 1 agency HCA next to him. He sits at the staff desk at the entrance to bay 4 at the end of the ward and sorts out piles of papers and dressings in their packets. He seems so much happier and relaxed now he is occupied. He picks up the packets and sorts them into different piles. The 1 to 1 HCA is standing next to him leaning against the wall next to him and quietly leans over and takes the packets of dressings from him and puts them back where they are usually stored on a mobile stand. Rock the Casbah is playing quietly in the background.

(Site 2 day 1)

The above illustrates ways in which the established racial presentation of Black people as dangerous, leads to privileges (McIntosh 1990) for White men in the same institutions. While a Black patient is assessed as at risk of violent behaviour, and subject to close security guard supervision, a White man with a similar risk assessment (he has a one‐to‐one agency HCA assigned to him) is allowed to walk around the ward and sit at a nurses desk at the entrance to the bay. In many ways this points to ways in which institutional racism in this context brings to the fore questions relating to how patients of colour can be perceived as being the ‘other’ (Jensen 2011), dangerous (Jackson 2020), out of place, or not belonging in these settings and thus in need of close supervision and control. As Jensen (2011) observes, this way of locating people leads to classifications of the deserving and undeserving patients.

### 2.6 Intersections of Dementia, Ethnicity, and Social Class

In addition to the intersections of dementia and ethnicity above, people living with dementia were often subject to class prejudice within these wards. It is widely accepted that social class has an impact on material conditions and health outcomes, with people from working class communities experiencing poor health outcomes (Nazroo 2003; Stopforth et al. 2023). In this paper we argue that in addition to these material conditions, it is important to consider how wider social attitudes to class impact on people's experiences of and access to care. Being categorised as belonging to a particular social class shaped the privileges available to a person and ward staff attitudes to them.

During a later shift/day of observation within the same general medical ward, a White man, Edward, is very frustrated at being contained within his single occupancy room by the one‐to‐one agency HCA assigned to him. Edward is identified and categorised as a White working‐class man. This is significant because while staff used security to manage perceived risks presented by John, a Black patient, the White patient is equally contained, but with routine agency HCA staff, despite his verbal threats of violence. The HCA is sitting just outside his open door, keeping him within the room. She is composed and calm, but does not look at him, and faces away from him into the ward, which appears to increase his frustration and distress.

Edward IF I CAN’T KILL THAT MAN AND MY WIFE, I AM IN THE SHIT. I wish I was bombing again, I would bomb this place to smithereens

HCA you would kill lots of innocent people?

She uses a very calm tone.

Edward Who is the boss?

HCA I will tell her you want to see her

Edward No there is no point.

He sounds very despondent and stands up and uses his walking frame to stand at the doorway. He is dressed in brown trousers and a brown cardigan over a shirt.

(site 2 day 14)

He shouts (in the section above and below) about killing his wife and bombing ‘this place to smithereens’, but also his frustration and anxieties about his missing dentures. The ward team say they will need to talk to ‘the doctors’ and as the afternoon progresses, he becomes very despondent. Of interest here is that staff take no action (there is also no action in relation to his dentures) and provide continued one‐to‐one support. Contrast this with the categorisation of the Black man, John in the above section, who was described as ‘unpredictable’, ‘aggressive’ and a ‘big man’ but was not verbally threatening violence.

In response to Edward’s frustrations, the Nurse in Charge goes over to him, smiles and laughs ‘Are you going to teach me the dirty word again?’

Edward is very angry and clearly frustrated: I am sitting here like a stuffed prune, I am sitting here without my teeth, why can’t I go to the dentist

RN Oh well we will ask the doctors for that.

The smiths play in the background ‘I want to go out tonight, but I haven’t got a stitch to wear…’

(Site 2 day 14)

Ward staff were acutely aware of the impact of these interventions. They described the use of legal frameworks (Deprivation of Liberty Safeguards (DoLS)) associated with restricting a person ‘you can't stop them’. However, this is only in relation to their impact on the middle‐class White patient David (who we met above), who is identified by staff as from a professional social class, indicated by his demeanour and the row of pens in his pyjama pocket, who received a number of privileges. See field notes below.

David has walked along the corridor and is now back at the nurses desk at bay 4. He is looking through one of the patient folders and reading it. The one‐to‐one moves him away and they walk down the corridor and come over to greet me.

HCA He is very active as you know, patients with dementia are very active, and their cognition can go up or down.

He puts an arm gently around David’s shoulders in a friendly embrace.

HCA He thinks he is at work, so he is a lot of work.

He gently guides him back to the bed and they sit together at the bedside.

HCA Because of deprivation of liberty, you have to be careful, you can't stop them, so I gently guide him back.

(site 2 day 14)

It is important here to highlight the ways in which the White middle class professional man's actions are rationalised, presented as normal, and supported. While the presence and actions of working class White and Black patients are met with a stricter differentiated culture of control (Garland 2002). In pointing to these hierarchies of control, even between these working‐class Black and White patients, the Black person is subject to stricter regimes of control.

The experience of their admission and of being contained impacted both patients. David and Edward are both frustrated and talk about their desire to leave the ward. Importantly, with Edward who is working class, the ward team do not respond to his frustration about being contained in his room. In contrast, the ward team below listen to David and provide reassurance about why he needs to stay in the ward. They ask him about his life experiences as a means of distraction. Meanwhile one of the team leave this encounter to admonish another patient (identity not known) in the wider ward to remain in bed.

The one‐to‐one is with David as he walks down the corridor, holding a collection of ward leaflets and talks to the team at the nurses station.

David ‘The doctor took my licence, the doctor said the driving was bad for your brain, so…’ He shrugs ‘Someone came to me this morning and said I will do you in half an hour, you know they stop you!’ He looks very indignant about his treatment and suddenly very angry.

The HCA one‐to‐one introduces David to the Nurse in Charge who is at the nurses station updating the rosters. She says to him: Did you use computers?

David Not really

RN Where did you work?

He talks about work, and about business taxation

Meanwhile the one‐to‐one HCA in the bay opposite watches over a patient in bed.

HCA Don't move please! She goes over to him and asks him to stay in bed.

(site 2 day 14)

Edward restricted to his single occupancy room is increasingly angry and frustrated. His cries of ‘I WANT TO GET OUT OF HERE’ become part of the ward soundscape; however, this has an impact on others. The middle‐class White man, David, now urgently wants to leave the ward and reach his car. In response he receives significant reassurance from the ward team. The cleaner removing bags of waste also reassures him, she smiles and says hello and appears to recognise his social class, by suggesting he drinks cocktails.

Edward ‘I WANT TO GET OUT OF HERE!!’

David hears this and looks startled, asks

What’s wrong with him?

HCA Nothing, he just wants to leave here

David heads down the corridor: I want to go to my car. he sounds very urgent

HCA Your car is ok

David why can't I do it

HCA it's all locked up and its fine don't worry about it

David I want to get out.

The HCA leads him back to his bedside, but he leaves and looks increasingly distressed. He passes the cleaner who is emptying the bins in the corridor‐ she smiles at him and says hello and they chat

Cleaner You just need a cocktail!

They laugh together and he walks to the day room and back again.

(site 2 day 14)

It is important to point out here the hierarchy in privilege, the levels of containment employed, and their impacts on these men. A White middle‐class man is allowed to move about the ward while a White working‐class man with overt expressions of violence is restricted to his room, both clearly articulate that they urgently want to leave the ward and are supervised by one‐to‐one HCAs. Meanwhile a Black man who has not expressed any verbal threats of violence is ‘othered’ (Jensen 2011), categorised as aggressive (Monk 2022), and is assigned a security guard.

### 2.7 Intersections of Dementia, Social Class, and Gender

In the previous sections we have shown how ethnicity and social class intersect to shape the privilege (McIntosh 1990) patients within these wards were accorded. We have shown the hierarch with regards to the nature and privileges or lack thereof of the measures put in place to control and accord (Garland and Gaughan 2002) patient movements within the ward. In this section we take a step further to show the role of gender in these interactions.

Female patients particularly those whose presentation was middle‐class were more likely to be given privileges (McIntosh 1990; Walkerdine 2021) within these wards than other patients. We explore in detail observations from one shift/day within a general medical ward, within a different hospital, where we can see these privileges in action. We examine the experiences of Amanda, a middle‐class White woman living with dementia, who has the demeanour and spirit of an actress and performer. The ward team locate her social class by referring to the professions of her husband and adult middle‐aged children. She is very elegant, dressed in co‐ordinated loungewear and sings as she walks around the bay and the wider ward. As she does this, she is greeted by members of the ward team and is permitted to go to the bedsides of other patients and to talk and sing to them. However, in the next bed, is Kathryn a working‐class White woman, also living with dementia. She has a distinct regional accent, is dressed in a blouse, jumper, and stay‐pressed trousers, and experiences significantly less privileges within this ward, which intensified over her admission. Every time she stands and attempts to leave her bedside, the ward team focus on returning her to sit in her bedside chair bed. She is further constrained by a ‘chair alarm’ [author], which sets off a loud blaring alarm when she stands, alerting staff to her attempts to walk from the bedside.

While social class plays a role in these interactions, it is further shaped by the gendered nature of the support they receive from ward staff. While there is a difference in the care they receive based on their social class, there is also a difference in the interactions ward staff had with them. The working‐class patients also experienced measures to control their movement. While a male working‐class patient (Edward, described above) was confined to his room with a one‐to‐one healthcare support, Kathryn, a female working class patient has a chair alarm to monitor and control her movements and contain her at the bedside. It appears staff may consider male Black and White working‐class patients as requiring close control. While female working class patients may be viewed as a potential disruption to the work of the ward.

In this bay of six women, Kathryn a White woman is sitting in the bedside chair, she stands and sets off the alarm attached to the chair, and she sits down again. The alarm has to be re‐set by one of the team and only then stops.

The white middle‐class woman in the next bed, Amanda, is singing, with Kathryn joining in across the bed and they sing together.

Kathryn stands up again from the chair, the alarm blares loudly, and she sits down again.

(site 5 day 1)

While both Amanda and Kathryn like to walk around the ward, it is the working‐class woman, Kathryn, who is controlled with a chair alarm, while Amanda is permitted to walk, and is encouraged to sing and interact with ward staff and patients.

The HCA is now with Amanda who gets up and walks from the bedside to the end of the bay and down the corridor using her walking frame and sings as she goes. The HCA is with her, and they stop at the nurses station and the team all chat and laugh with her as she sings loudly and tunefully ‘oh what a wonderful morning, oh what a wonderful day…’ She has a very ‘BBC English’ ‘received pronunciation’ voice from the 1950’s, she also uses lots of expressive and sweeping arm actions in time with the song as she sings. Amanda continues walking along the ward, the HCA encourages her, and everyone smiles and says hello to her as they pass. She stops at each bay in turn and sings to the other patients. Using her walking frame, she walks through the ward to the exit and blows kisses to all the patients in bay 3 as she passes.

(site 5 day 1)

Kathryn and Amanda both use walking frames; however, one is able to walk about the ward, while the other, is contained and is initially not permitted to leave her bedside. The HCA additionally takes hold of Kathryn's walking frame to stop her from walking further and turns it around. This form of control is clearly highly distressing for Kathryn.

Kathryn and Amanda are both getting up from their bedside chairs to walk. Amanda uses her frame to walk out of the bay. The HCAs gives Kathryn a frame from another bedside and stands close to her and when she reaches the end of her bed, turns her around and leads her back to the bedside.

Kathryn LEAVE ME ALONE

She is very angry and wants to leave her bedside

HCA 121 I can't leave you alone‐holding onto her walking frame with both hands

Kathryn turns and attempts to leave her bedside and the HCA one‐to‐one stays with her as she goes. She turns to her sharply and says: ‘LEAVE ME ALONE!’

HCA I can't leave you alone

Of note above, is that staff talk to Kathryn in very short statements, emphasising their authority and power ‘I can't leave you alone’ and contracted instructions ‘turn around’ (below), while staff talk to Amanda is far more conversational in nature. The intensification of control measures has affective impacts on patients living with dementia—it results in a visceral response, the demand to be left alone. This continues and increases to an irretrievable breach in relationships.

At the same time, Kathryn uses the walking frame to walk out of the bay and into the corridor

HCA You need to go back to your bedside

Kathryn DON’T TELL ME WHAT TO DO NO ONE CAN TELL ME WHAT TO DO

HCA Well, you need to go back to bed

Kathryn marches to the end of the bay and tries to open the double doors that lead out of the ward (this is locked). The HCAs follow her and tries to turn her around by holding and steering the walking frame around to face the other way

Kathryn NO LEAVE ME ALONE GET YOUR HANDS OFF ME

HCA Turn around!

Kathryn Have a good laugh, leave me alone, LEAVE ME ALONE. Kathryn is now very distressed.

Meanwhile, Amanda is walking along the bay using her frame across the bay to reach the woman in bed 1.

Amanda Can I sing to her? Maybe I should sing my special song. she starts to sing ‘You are my hearts delight…’

The HCA steers her away from the bedside and they walk along the main ward corridor together. Amanda stops and sings at the nurses station and all the staff says hello and are very friendly and warm in their response to her. She moves on and stops at each of the bays and sings to other patients.

(Site 5 day 1)

Amanda a middle‐class woman is tolerated as she continues to sing and walks freely around the bay and wider ward. She is greeted by the ward team and is given the privilege of talking and singing to staff and patients. However, Kathryn faces significant controls. When she stands and uses her frame to walk, the bay team attempt to control her and return her to her bedside.

This tension between managing perceived disruptive patients along lines of social class, ethnicity, and gender (Nash 2008), occurred throughout these shifts. White male and female middle‐class patients were observed to receive more privileges within these wards than working class patients (Walkerdine 2021). Meanwhile Black male patients who has not expressed verbal aggression may present risk to staff by virtue of their ethnicity and are categorised as ‘aggressive’ and ‘big man’ (Jackson 2020). The surveillance (Foucault 2016) patients are subject to across these wards results in increased frustration, anger, and high levels of distress.

## 3 Conclusion/Discussion

Drawing on concerns for inclusion and equality and the need to improve the care people living with dementia (Driessen and Ibanez 2020; Haeusermann 2018; Scales et al. 2017) receive in acute hospital settings as a point of departure, this paper highlights the significance of intersectionality (Crenshaw 1991) as both a theoretical and methodological approach in dementia research. To date, research has rarely paid attention to how intersections and power relations influence care and health outcomes for people with intersecting identities living with dementia. This paper has shown how care is contoured by the wider organisational and sociopolitical milieu. Specifically, we have illustrated how the normalisation of racial, social class and gender, categories create hierarchies, which privileges some patients to the detriment of others. Crenshaw (1991), hooks (2000) and Nash (2011) argue that power relations in everyday interactions have been employed to show how ethnicity, social class and gender intersects to bring about differentiated approaches to privileges for people living with dementia in acute hospital settings.

We have drawn and extended the application of Garland's (2002) concept of ‘cultures of control’ to show how in a fast‐paced acute hospital ward environment, organisational priorities inform the need to control patients with the view to maintaining order and sustain the timetabled work of the ward (Featherstone and Northcott 2020). While Garland's concept emanates from his observations of criminal justice systems, his view that contemporary society has presented new challenges to the justice system and led to a culture of control is key.

We argue that increasing numbers of people living with dementia admitted to acute hospital settings equally present a challenge to contemporary healthcare provision. The challenge is not just the widely publicised pejorative term of “bed blocking” (Gaughan, Gravelle, and Siciliani 2015), but the care of those perceived to disrupt the embedded organisational timetables of the institution who do not reflect expectations of an ideal compliant patient, and who may require controlling. In this context, we have shown how organisational measures of control including one‐to‐one care, use of security guards, and verbal commands, are shaped by implicit racist, classist and gendered attitudes about who deserves intensified control or the privilege to walk and interact with others within these wards. We have shown how older African and Caribbean patients are likely to be subject to more control and presented as a threat requiring heightened security supervision, whereas White patients receiving the same categorisation were assigned enhanced care. We have further illustrated how these attitudes extend to gender, with female patients subject to softer regimes of control but differentiated by class. White middle‐class male and female patients were given more privileges than their working‐class counterparts. The impact of these differentiated approaches to privileges is increased distress and poor experiences of care for marginalised patients who are particularly vulnerable and at risk of poor outcomes, with the potential to impact negatively on both their dementia and their admitting condition and lead to further distress and deterioration. As these institutional classifications of a person (for example as ‘aggressive’) and the measures to control them (use of one‐to‐one care and security guards in their care) enter an individual's medical records, they in turn reduce opportunities for discharge and to return to their home and increase their risk of institutionalisation. Consequently, these practices reinforce cultures of care that buttress inequalities in health experiences and outcomes.

Therefore, reclaiming the political implications of intersectionality and the visceral consequences of multiple layers of discrimination, we emphasise the significance of intersectionality not merely as an intermingling of a number of innocuous factors and individual characteristics, but rather imbued with power and the social situatedness of encounters in which care is provided. As Foucault 2003 and Butler 2002 observe, the exertion of power on bodies should be understood as an insidious political tool; we add that it is at once invisible and normalised, with significant consequences and negative impacts for patients with intersectional identities. We also suggest that this has negative impacts for staff; the organisation and delivery of care in these institutional settings reflects ‘the way we do things here’, which they do not have the power to challenge (Featherstone and Northcott 2020). Therefore, we emphasis the use of intersectionality, which has at its analytical core a focus on political and power relations that coalesce in the social encounters within care settings. Eliding this renders intersectionality a mere concept.

In conclusion our paper demonstrates how age and living with dementia in acute hospital settings intersects with ethnicity, social class and gender to bring about the routinisation of inequality. In this context, people living with dementia of colour and who are working class face intensified levels of control, while White middle‐class patients are given privileges to roam and interact with others on the ward. We argue that the intensification of these controls and surveillance on marginalised patients impacts their experiences of care. The paper has highlighted the affective impacts (Fanon 2021) of heightened control, surveillance and containment for people living with dementia admitted to these wards.

This is in keeping with Goffman's ((1961) 1991;41) concept of ‘looping’ as a process within the total institution in which the organisation adopts a defensive to maintain its operations in the face of new inmates deemed disruptive. Meanwhile the inmate sees this response as an attack. We have illustrated these characteristics in the care of people living with dementia in these wards. People living with dementia within these wards responded to the organisational culture of timetabled bedside care by resisting and behaving in ways perceived by the ward as disruptive, and inappropriate. In response the organisation intensifies control, generating further distress in the person, which leads, in turn, to further tightening, containment, and so on (Featherstone and Northcott 2020). These institutional cultures of control reinforced and normalised racist, classist and gendered views of people living with dementia which perpetuates inequality and discrimination.

We have demonstrated through our detailed ethnographic data and analysis ways to understand to the social world of these wards, and how this connects with wider issues of care, control and subjugation within institutional settings (Glaser and Strauss 1967; Saunders et al. 2018). The institutional racism and attitudes to gender, social class and ageing, and the ways in which they permeate the routine organisation and delivery of care within these NHS acute hospital wards significantly impact people living with dementia, and in turn, increases the consideration of care pathways that emphasise their discharge to institutional settings.

There is need for health and social care institutions to recognise and respond to the power relations that influence the care and health outcomes of people with intersecting identities living with dementia and for further research utilising intersectionality (Nash 2011) as both a theoretical and methodological approach in dementia research, utilising primary data to identify the experiences of vulnerable and marginalised older people across health and social care settings to inform interventions to improve care practices.

## Author Contributions

**Katie Featherstone:** conceptualisation (equal); data curation (lead); formal analysis (lead); funding acquisition (lead); investigation (lead); methodology (lead); project administration (lead); supervision (lead); writing–original draft (equal); writing–review & editing (equal). **Shadreck Mwale:** conceptualisation (lead); data curation (equal); formal analysis (equal); writing–original draft (lead); writing–review and editing (lead). **Andy Northcott:** conceptualisation (equal); data curation (equal); formal analysis (equal); writing–original draft (lead); writing–review and editing (equal).

## Data Availability

The data that support the findings of this study are available on request from the corresponding author. The data are not publicly available due to privacy or ethical restrictions.
